# Predictors of Survival in Subtotally Resected WHO Grade I Skull Base Meningiomas

**DOI:** 10.3390/cancers13061451

**Published:** 2021-03-22

**Authors:** Michele Da Broi, Paola Borrelli, Torstein R. Meling

**Affiliations:** 1 Faculty of Medicine, University of Oslo, 0372 Oslo, Norway; Michele.dabroi@studenti.univr.it; 2 Department of Neurosurgery, Oslo University Hospital, 0372 Oslo, Norway; 3 Department of Medical, Oral, and Biotechnological Sciences, Laboratory of Biostatistics, University “G. d’Annunzio” Chieti-Pescara, 66100 Chieti, Italy; paola.borrelli@unich.it; 4 Department of Clinical Neurosciences, Division of Neurosurgery, Geneva University Hospitals, 1205 Geneva, Switzerland; 5 Faculty of Medicine, University of Geneva, 1206 Geneva, Switzerland

**Keywords:** surgery, neurosurgery, brain tumor, intracranial tumor, meningioma, overall survival, skull base meningioma, subtotal resection

## Abstract

**Simple Summary:**

Radical excision of meningiomas of the skull base has always been a major surgical challenge because of the complex location and the risk of neurovascular damage related to it. In these cases, the benefits of gross-total resection must be balanced with the quality of life after surgery. In the present study, we investigated a cohort of 212 consecutive patients who underwent partial resection of a benign skull base meningioma in order to find predictors of overall survival (OS). Moreover, we analyzed the clinical outcomes and cases of retreatment for progressive disease. In our case series, advanced age at surgery and a preoperative Karnofsky performance status of <70 were negative predictors of OS. Patients who underwent further procedures did not have reduced OS. Overall, surgical and neurological outcomes of STR skull base meningiomas were worse compared to case series, including also completely resected tumors.

**Abstract:**

Background: Although gross total resection (GTR) is the goal in meningioma surgery, this can sometimes be difficult to achieve in skull base meningiomas. We analyzed clinical outcomes and predictors of survival for subtotally resected benign meningiomas. Methods: A total of 212 consecutive patients who underwent subtotal resection (STR) for benign skull base meningioma between 1990–2010 were investigated. Results: Median age was 57.7 [IQR 18.8] years, median preoperative Karnofsky performance status (KPS) was 80.0 [IQR 20.0], 75 patients (35.4%) had posterior fossa meningioma. After a median follow-up of 6.2 [IQR 7.9] years, retreatment (either radiotherapy or repeated surgery) rate was 16% at 1-year, 27% at 3-years, 34% at 5-years, and 38% at 10-years. Ten patients (4.7%) died perioperatively, 9 (3.5%) had postoperative hematomas, and 2 (0.8%) had postoperative infections. Neurological outcome at final visit was improved/stable in 122 patients (70%). Multivariable analysis identified advanced age and preoperative KPS < 70 as negative predictors for overall survival (OS). Patients who underwent retreatment had no significant reduction of OS. Conclusions: Advanced age and preoperative KPS were independent predictors of OS. Retreatments did not prolong nor shorten the OS. Clinical outcomes in STR skull base meningiomas were generally worse compared to cohorts with high rates of GTR.

## 1. Introduction

Radical excision of meningiomas located at the skull base has always been a major surgical challenge [1]. Complex location with proximity to and/or encasement of neurovascular structures, large size, and hard tumor consistency may represent insurmountable obstacles that impede gross-total resection (GTR). On the one hand, subtotal resection (STR) often leads to progression [2] and subsequently often to new invasive procedures [3]. Consequently, GTR has always been considered the aim to pursue [1,3,4,5,6,7,8,9]. On the other hand, the increasing mean age of patients with meningiomas [6,10,11] imposes less aggressive surgical interventions and recourse to adjuvant radiotherapy (RT) or stereotactic radiosurgery [7,12,13,14].

Besides patient age and preoperative clinical conditions, the benefits of GTR must be balanced with the quality of life after surgery [7]. Schneider et al. [15,16] showed in two recent publications that Simpson grade I resection in posterior fossa meningiomas and fronto-basal meningiomas were significantly associated with higher cranial nerve morbidity and CSF leakage rates as compared to less aggressive excisions. In light of these reports, the prospect of a steady increase of STR skull base cases appears tangible.

At present, only a few studies focus on the clinical outcome of STR skull base meningiomas. In 1994, Goldsmith et al. [17] published a retrospective analysis of 140 STR meningiomas treated with postoperative RT. It was one of the first publications to provide evidence in favor of adjuvant RT and describing long-term overall survival (OS) and progression-free survival (PFS) after partial resections of meningiomas. In 1996, Mathiesen et al. [18] presented a historical cohort (surgical period 1947–1982) of skull base meningiomas with complete long follow-up (mean: 18 years). In Mathiesen’s study, only a minority of Simpson grade IV and V resections remained stable, whereas the rest showed progressive symptoms. Nearly 61% of STR patients died as a result of the tumor, usually within 10 years from the first operation, and none of the patients followed for over 20 years were free from symptoms of progression. More recently, Materi et al. [19] identified preoperative tumor volume, falcine, and tentorial locations, and African American race as risk factors of recurrence after STR of meningiomas.

Given the current lack of reports on survival in partially resected meningiomas, we wanted to use a large retrospective cohort to identify predictors of OS and retreatment-free survival after Simpson grade IV and V resection of World Health Organization (WHO) grade I skull base meningiomas.

## 2. Results

### 2.1. Overall Characteristics

In this case series, 212 consecutive patients who underwent STR of intracranial WHO grade I skull base meningioma were investigated. A total of 259 craniotomies were performed in the time frame 1990–2010. The Median follow-up duration was 6.2 [IQR 7.9] years, and no patient was lost to follow-up. The female-to-male ratio was 3.6. The median age at primary surgery was 57.7 [IQR 18.8] years. Preoperative KPS was over 70 in 156 patients (73.6%), and median Karnofsky performance status (KPS) was 80.0 [IQR 20.0]. Indications for surgery were based on patient’s symptoms in 98.6% of cases (*n* = 209), whereas 3 patients (1.4%) underwent surgery due to increased size documented radiographically. Preoperative symptoms were seizure in 28 patients (13.2%), raised intracranial pressure (ICP) in 66 patients (31.1%), and neurological deficits in 154 patients (72.6%). One hundred thirty-seven meningiomas (64.6%) were located in the anterior skull base region, whereas 75 (35.4%) were in the posterior fossa. The specific location of each lesion is listed in Table 1. Almost all patients underwent Simpson grade IV resections (*n* = 207, 97.6%), while only 5 excisions (2.4%) were classified as Simpson grade V.

### 2.2. Clinical Outcomes

**Figure 1:**
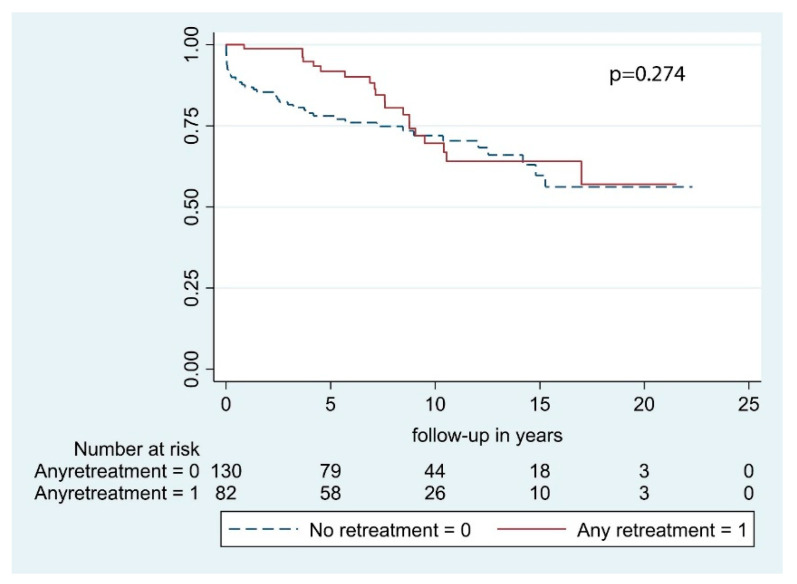
Overall survival by retreatment.

Neurological outcome at final visit was improved or remained stable compared to the preoperative status in 70.1% of cases (*n* = 122), while neurological status had worsened in 29.9% of patients (*n* = 52). With regard to complications after surgery, 9 postoperative hematomas (3.5%) and 2 postoperative infections (0.8%) occurred during the whole study period. Ten patients died within 30 days from surgery (4.7%). Retreatment rate at 1-year was 16.0% (*n* = 34), at 2-years was 23.1% (*n* = 49), at 3-years was 26.9% (*n* = 57), at 5-years was 33.5% (*n* = 71), at 10-years was 37.7% (*n* = 80), and at 15-years was 38.2% (*n* = 81) (Table 2). In our case series, 33 patients (15.6%) underwent 2 surgical procedures, 3 patients (1.4%) underwent 3 resections, and only 2 patients underwent respectively 4 and 5 tumor excisions. After a second surgery, no histological transformation of meningiomas occurred, whereas, after the third procedure, one atypical lesion was found. The median time-to-retreatment between the primary procedure and the second one was 3.1 [IQR 3.2] years, while between the second and the third one was 3.0 [IQR 2.9] years. Further analyses were not carried out due to the paucity of the sample. Regarding radiation therapy, 8 patients (3.8%) received adjuvant RT, of whom 2 by means of conventional fractionated RT, while the other 6 via stereotactic radiosurgery (SRS). Patients who underwent retreatment had a minimal insignificant prolongation of OS compared to those who did not undergo further operative procedures (7.6 years [IQR 7.6] vs. 7.2 years [IQR 8.58], *p* = 0.295) (Table 3; Figure 1). More specifically, median OS in patients who underwent second surgery without adjuvant RT was 10.6 [IQR 6.4] years, in patients retreated via RT alone OS was 5.4 [IQR 5.2] years, and in those who underwent retreatment with both surgery and adjuvant RT, median OS was 9.7 [IQR 5.7] years, and the difference was not statistically significant (*p* = 0.175).

### 2.3. Predictors of Overall Survival

In the present cohort, the OS at 1-year was 91.5%, at 2-years was 90.6%, at 3-years was 88.2%, at 5-years was 83.4%, at 10-year was 71.0%, and at 15-years was 61.3%. Regarding retreatment-free survival, it was 75.9% at 1-year, 67.9% at 2-years, 61.7% at 3-years, 52.4% at 5-years, 42.4% at 10-years, and 34.6% at 15-years.

**Figure 2:**
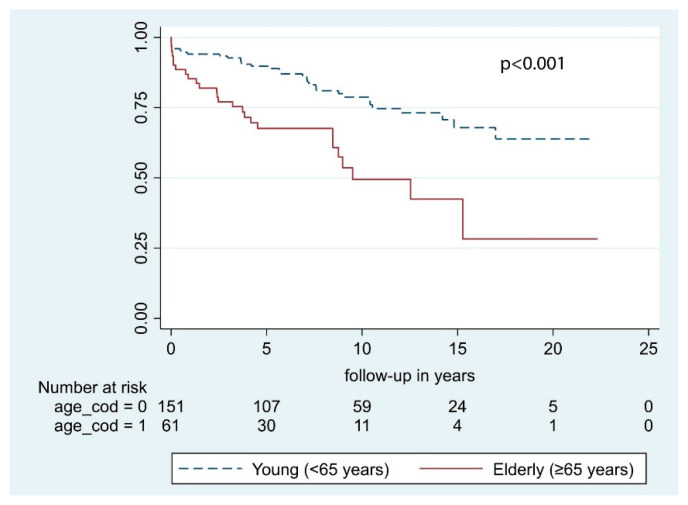
Overall survival by age at primary surgery.

**Figure 3:**
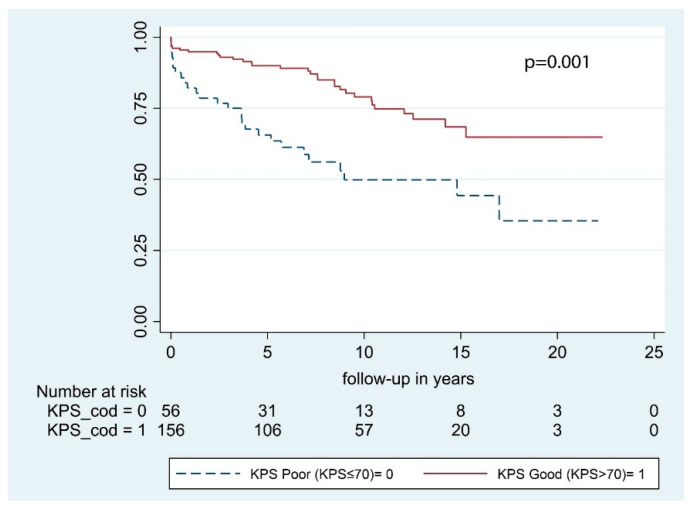
Overall survival by preoperative KPS.

According to our univariate analysis, the following predictive factors were significant for OS: Sex, age at primary surgery, and preoperative KPS. Conversely, neurological deficits at presentation, tumor location, and bony invasion had no significant impact on OS (Table 3). In multivariable analysis, only age at primary surgery and KPS were significant with *p* <0.001 and *p* = 0.001, respectively (Figure 2 and Figure 3). According to multivariable analysis, patients with poor KPS had more than 6 times the risk of developing a postoperative hematoma requiring surgical evacuation (OR = 6.2 [1.1–34.6], *p* = 0.039), whereas no significant correlation with worsened neurological outcome (*p* = 0.182), postoperative infections (*p* = 0.987), or 30-day mortality (*p* = 0.318) was found.

## 3. Discussion

In the present study, a cohort of 212 subtotally resected WHO grade I skull base meningiomas was investigated to detect predictors of survival and analyze clinical outcomes. STR was defined as Simpson grade IV or V resections, as described by the European association of neuro-oncology (EANO) [5].

### 3.1. Clinical Predictors of Survival

Our results show that age at surgery, both dichotomized at 65 years of age and considered as a continuous variable, and preoperative KPS ≤ 70 were independently correlated with a reduced OS (Table 3). As expected, younger patients had significantly longer OS after STR compared to the older individuals (Figure 2). Specifically, every year carries a 5% increased risk of death. In the present study, only WHO grade I meningiomas were included, and OS was calculated considering all causes of death. Therefore, the effect of different residual life expectancies (RLE) cannot be ruled out. Considering patients >65 years, the median age at surgery was 71.7 [IQR 7.3] years and median OS was 5.0 [IQR 5.9] years, the sum of which was quite similar to the average life expectancy in Norway at 77.3 years in 1990 [20]. Conversely, the median age in the younger cohort (<65 years) was 52.0 years [IQR 14.9] years, and median OS was 7.9 years [IQR 8.7] years. In this case, it is likely that median OS mirrors the length of follow-up instead of RLE. Our conjectures are shared by other authors [21,22,23]. Among them, Hasseleid et al. [3] found that increased median age at diagnosis was independently correlated with a reduction of OS and that this observation was biased by the naturally limited RLE of older individuals. Later, Brokinkel et al. [21] found that the OS in older patients was significantly shorter as compared to the sex- and age-matched population, indicating excess mortality [24]. Altogether, the difference detected in our cohort is likely ascribable to different RLEs. However, the decision of operating on elderly individuals should always be cautiously weighed up because of the increased risk of postoperative hemorrhages, wound, or systemic infections [25,26,27].

An independent association between KPS > 70 and prolonged OS was identified (Table 3; Figure 3). Indeed, patients with a good preoperative KPS had less than half the risk of death of any cause during the study period compared to those with poorer KPS. We further analyzed the predictive role of KPS with regard to neurological outcome, early postoperative complications, and perioperative mortality. According to multivariable analysis, patients with poor KPS had more than 6 times the risk of developing a postoperative hematoma requiring surgical evacuation. Preoperative KPS is a robust predictor of survival in large meningioma cohorts [4,28,29], as well as in more selected subgroups and is included in several prognostic scores [10,30,31,32]. However, KPS reflects patient’s general condition and provides no information to clinicians with respect to neurological and surgical outcome. The inverse correlation between KPS and postoperative hematomas delineated in the present study is limited by the small number of patients analyzed and should be further investigated in a prospective study.

### 3.2. Clinical Outcomes

Considering the skull base meningioma series published during the last 30 years, the rate of postoperative hematomas ranged from 0.0% reported by Samii et al. [33] to 3.4% extra-axial and 5.4% intracerebral hematomas presented by Levine et al. [34]. Meling et al. [11] identified 5.3% postoperative hematomas requiring surgery in a cohort of elderly patients (over 70 years). The postoperative hematoma rate recorded in the present study was 3.5%, which is in line with the literature [1,27,35]. With respect to postoperative infections, our rate was 0.8% (Table 2). This is also in line with previous reports [1,11,33,34], which have ranged from 0.0% described by Roser et al. [27] in patients younger than 65 years to 7.0% reported by the same authors for patients over the age of 65. The 30-day mortality rates reported in the literature ranged from 0.0% to 2.0% [27,34]. Early postoperative complications identified in our cohort are comparable with other high GTR rates series. On the other hand, the perioperative mortality detected in the present study was 4.7%, which is twice as high as most publications on skull base meningiomas describe [1,11,33] (Table 2). We wanted to investigate whether there was an association between 30-day mortality and the clinical variable considered in this study. In the univariate analysis, reoperation for early postoperative hematoma, seizure, or symptoms of raised ICP was significant. However, according to our multivariable analysis, only seizures at presentation were significantly associated with death within 30 days from surgery (OR = 12.0 [2.6–62.6], *p* < 0.01).

Data on neurological outcomes after surgery were available only in few skull base meningioma series. Therefore, the comparison with our results is limited. Considering case series of adult and elderly patients, the rates of worsened neurological outcomes range from 7% to 17% [1,11,27]. In the present study, 30% of patients had worsened neurological outcomes at the final visit, which is more than twice as high as the series that included all Simpson grades (Table 2). In absolute terms, the rate of worsened neurological outcomes reported here is considerable. This result indicates that in demanding skull base meningiomas, surgery entails a tangible risk of worsening patients’ neurological status and quality of life. However, it may also mirror the underreporting of neurological deterioration in some past case series. The rationale for operative management can be found by analyzing the whole picture. In the current series, 72.6% of patients had neurological deficits at presentation, and at the final visit, 70.1% of them had improved or stable neurological outcomes (Table 1).

Overall, our results show complication and morbidity rates comparable or even worse than those reported in other case series that included GTR meningiomas. To some extent, this is a countertrend compared with the belief that STR will be associated with better postoperative status compared to radical excisions. However, in order to better investigate the benefits or disadvantages of function sparing resection, the evaluation of the quality of life scales should be included. Unfortunately, the retrospective analysis of our clinical records did not allow us to extract these data. We hope that the present study could pique the interest in this topic and further analyses may shed light on the impact of STR on quality of life of the patients carrying meningiomas in complex locations.

### 3.3. Tumor Location

For statistical purposes, meningiomas in the present study were dichotomized into posterior fossa and fronto-basal location. As reported by some authors [4,36], posterior fossa meningiomas constitute a subset of intracranial meningiomas because of their peculiar location, different clinical presentation, and behavior. For these reasons, GTR rates are usually lower in comparison to other locations [15]. According to our analysis, posterior fossa meningiomas had comparable OS after STR to their fronto-basal counterpart (Table 3). These findings indicate that location and all its implications do not influence the OS in any way in skull base meningiomas after partial excision. Preservation of quality of life should always be paramount when surgeons approach these difficult lesions, and heroic attempts to achieve more radical resections should be avoided.

### 3.4. Retreatment and Radiation Therapy

One of the major problems associated with STR meningiomas is the growth of residual tumors. As demonstrated by Simpson in 1957 [2] and successively by many other authors [3,37,38], the radicality of resection is inversely related to the frequency of a relapse. The analysis of Kaplan–Meier curves demonstrates an initial split. In the first 7 years, patients who underwent retreatment had a better OS. Thereafter, the curves cross each other and on the whole, the OS is comparable (Figure 1). Anyway, the cases of progression that were managed operatively were selected based on the presence of symptoms and/or documentation of residual growth. Information about patients handled conservatively was not available. Hence, the effect of selection bias on OS cannot be ignored, and the generalizability of our results to a population of recurrent skull base meningiomas is limited.

Recent trends in neurosurgery have led to the concept of function sparing resection, where STR is undertaken, and treatment is completed with SRS or fractionated RT. This combined approach has become the standard treatment in many institutions and is supported by evidence reported by several authors [39,40,41]. Unfortunately, the number of patients in the present study treated in this fashion is very limited, namely 6 patients with SRS and 2 with fractionated RT. Due to the small size of our sample, further analyses on irradiated patients were not feasible. Therefore, the present paper cannot conclude anything about the role of RT in subtotally resected benign SBM. Future studies, ideally randomized control clinical trials, are needed to investigate this important issue.

## 4. Materials and Methods

### 4.1. Patient Cohort

A Norwegian population-based cohort of skull base meningiomas treated surgically between 1990 and 2010 at the Oslo University Hospital (OUH) was reviewed. OUH was a tertiary referral center consisting of 2 neurosurgical units (Rikshospitalet and Ullevaal) that captured all meningioma patients within an area of approximately 3 million inhabitants (56% of the Norwegian population). There were 212 consecutive WHO grade I subtotally resected lesions identified. The location of the tumors and residual after surgery were confirmed by post-contrast imaging studies. The age cut-off we used to define older individuals was set at 65 years of age based on the report from Ostrom et al. [42], which identified a dramatic increase of meningiomas’ incidence after age 65 years. The extent of resection (EOR) was assessed using the Simpson grade scale [2]. STR was defined as Simpson grade IV or V in accordance to the EANO definition [5]. RT performed within 90 days from primary surgery was considered adjuvant RT, while all procedures (both conventional fractionated RT and SRS) undertaken after 90 days were considered retreatment. The WHO grading system was used to classify the histology of meningiomas. Since the WHO criteria have changed during the time-frame of our study, all lesions underwent a formal reclassification process according to the 2016 WHO classification carried out by a neuropathologist [43]. No patient was lost to follow-up for the assessment of outcomes. Neurological status at the final visit was dichotomized into improved-stable or worsened compared to preoperative status. Any neurological status worse than preoperative status was considered deteriorated status. Early postoperative complications were defined as on-site hematoma or surgical site/deep infection requiring operative intervention and corresponding at least to grade IIb complication in the Landriel–Ibanez classification [44]. Since in the present cohort only STR meningiomas were included, relapses after primary surgery were considered regrowth. All residual tumor growth with radio-clinical correlations occurring at the site of the previous surgery were considered. Lesions occurring at locations other than the primary site of the tumor were excluded. In order to avoid subjectivity in differentiating postsurgical tumor remains from scars located near the resection sites, the time-to-retreatment was calculated as the time between primary surgery and the first subsequent procedure (either RT or a new operation). Indications for retreatment were symptomatic progression of the disease or asymptomatic growth of residual lesion documented on imaging. Radiological re-growth without clinical expression, thus not requiring any adjuvant treatment, were not considered. Vital status (alive or dead) and time of death was obtained from the Norwegian Population Registry (Folkeregisteret) on 21/01/2011.

### 4.2. Ethics

The study was regulated by the Personal Data Act/Personal Health Data Filing System Act and approved by the Data Protection Official, registered Norwegian institutional review board at OUH (2017/5204). Under this regulation, the patient’s written informed consent was not required to collect and analyze data.

### 4.3. Statistics

Descriptive analysis was carried out using median and IQR for the quantitative variables and percentages values for the qualitative ones. Normality distribution was assessed by the Shapiro–Wilk test. The association between variables was investigated by Pearson Chi-Squared or Fisher Exact. Multivariable analyses to investigate the association between preoperative KPS and clinical outcomes, 30-day mortality, and preoperative parameters were carried out using a logistic regression model (Odds Ratio—OR and 95%CI). Survival analysis was performed by applying the Kaplan–Meier estimator and Log-rank test for equality of survivor functions. The association with clinical features was analyzed with the Cox model of proportional hazards (Hazard Ratio—HR and 95%CI), and the applicability assumption was evaluated by the Schoenfeld test. Statistical significance was set at <0.05. All analyses and graphical drawing were performed using STATA^®^ software v15.1 (StataCorp, College Station, Texas, TX, USA).

## 5. Conclusions

In this cohort of subtotally resected WHO grade I skull base meningiomas, age at surgery and preoperative KPS were independent predictors of OS. Retreatments did not prolong nor shorten the OS. However, surgical and neurological outcomes in subtotally resected skull base meningiomas were generally worse compared to meningioma series with high rates of GTR.

### Strengths and Limitations of the Study

The strength of this study is the long and complete follow-up (median = 6.2 [IQR 7.9] years). This is the one of the largest case series that analyzes survival and surgical outcomes in STR meningiomas. The pre- and postoperative postcontrast imaging studies were reviewed to confirm tumor location and EOR. With respect to data quality, we only used end-points that were easily verifiable (i.e., 30-day mortality, reoperation for hematomas, and reoperations for infections).

However, this study is limited by its retrospective nature, despite data from 2003 being collected prospectively. For this reason, the cause of death was not available for every patient, and hence we cannot calculate the disease-specific survival, and the OS includes mortality due to any cause. Volumetric data on residual tumor growth after primary surgery, as well as the number of patients with re-growing lesions who did not receive retreatment, were not always complete. Hence, they were excluded from data collection. Moreover, the single-center design of the study limits greatly the generalizability of our results to other neurosurgical departments. Analysis on postoperative complications is limited to postoperative hematomas and on-site or deep infections, whereas medical complications, such as pulmonary embolism and minor complications that did not require surgery, were excluded from data collection.

## Figures and Tables

**Table 1:** Overall characteristics.

Parameter		*n*	%
		212	100
Sex			
	Male	46	21.7
	Female	166	78.3
Age at primary surgery			
	<40 years	24	11.3
	40–50 years	35	16.5
	50–60 years	64	30.2
	60–70 years	51	24.1
	≥70 years	38	17.9
Preoperative KPS			
	100	4	1.9
	90	72	34.0
	80	80	37.7
	≤70	56	26.4
Presenting symptoms			
	Seizure	28	13.2
	Raised ICP	66	31.1
	Neurological deficits	154	72.6
	Asymptomatic	3	1.4
Location			
	Supratentorial	3	1.4
	Infratentorial	21	9.9
	Tuberculum sellae/suprasellar	30	14.2
	Cavernous sinus	14	6.6
	Petroclival	27	12.7
	Intraorbital	13	6.1
	Olfactory groove	10	4.7
	Middle fossa/Cavum Meckeli	7	3.3
	Medial sphenoid wing	50	23.6
	Lateral sphenoid wing	10	4.7
	Craniocervical junction/Foramen magnum	6	2.8
	CP angle	21	9.9

CP angle: Cerebello-pontine angle; ICP: Intracranial pressure; KPS: Karnofsky performance status.

**Table 2:** Clinical outcomes.

Clinical outcome		*n*	%
**Surgical outcome**			
	Postop hematomas	9/259	3.5
	Postop infections	2/259	0.8
	30-day mortality	10/212	4.7
**Neurological outcome at final visit**			
	Improved-unchanged	122	70.1
	Worsened	52	29.9
**Retreatment rate**			
	1-year	34	16.0
	2-year	49	23.1
	3-year	57	26.9
	5-year	71	33.5
	10-year	80	37.7
	15-year	81	38.2

**Table 3:** Univariate and multivariable Cox model for overall survival.

Variable	Univariate Analysis		Multivariable Analysis	
	HR (95%CI)	*p* Value	HR (95%CI)	*p* Value
Sex M vs. F	1.70 (0.99–2.93)	0.052	1.24 (0.71–2.17)	0.446
Age at primary surgery years	1.05 (1.03–1.08)	<0.001	1.05 (1.02–1.07)	<0.001
Preoperative KPS Good vs. Poor	0.35 (0.21–0.59)	<0.001	0.42 (0.24–0.71)	0.001
Neurological deficits Yes vs. No	1.49 (0.80–2.77)	0.199	-	-
Location Posterior fossa vs. fronto-basal	0.89 (0.52–1.53)	0.692	-	-
Bony invasion Yes vs. No	0.61 (0.31–1.18)	0.143	-	-
Retreatment Any retreatment vs. No retreatment	0.74 (0.43–1.26)	0.274	-	-

## Data Availability

The datasets generated during and/or analyzed during the current study are available from the corresponding author on reasonable request.

## References

[1] Meling T.R., Da Broi M., Scheie D., Helseth E. (2019). Meningiomas: Skull base versus non-skull base. Neurosurg. Rev..

[2] Simpson D. (1957). The recurrence of intracranial meningiomas after surgical treatment. J. Neurol. Neurosurg. Psychiatry.

[3] Hasseleid B.F., Meling T.R., Ronning P., Scheie D., Helseth E. (2012). Surgery for convexity meningioma: Simpson Grade I resection as the goal: Clinical article. J. Neurosurg..

[4] Corniola M.V., Lemee J.M., Da Broi M., Joswig H., Schaller K., Helseth E., Meling T.R. (2019). Posterior fossa meningiomas: Perioperative predictors of extent of resection, overall survival and progression-free survival. Acta Neurochir..

[5] Goldbrunner R., Minniti G., Preusser M., Jenkinson M.D., Sallabanda K., Houdart E., von Deimling A., Stavrinou P., Lefranc F., Lund-Johansen M. (2016). EANO guidelines for the diagnosis and treatment of meningiomas. Lancet Oncol..

[6] Konglund A., Rogne S.G., Lund-Johansen M., Scheie D., Helseth E., Meling T.R. (2012). Outcome following surgery for intracranial meningiomas in the aging. Acta Neurol. Scand..

[7] Lemee J.M., Corniola M.V., Da Broi M., Joswig H., Scheie D., Schaller K., Helseth E., Meling T.R. (2019). Extent of Resection in Meningioma: Predictive Factors and Clinical Implications. Sci. Rep..

[8] Meling T.R., Da Broi M., Scheie D., Helseth E., Smoll N.R. (2019). Meningioma Surgery-Are We Making Progress?. World Neurosurg..

[9] Odegaard K.M., Helseth E., Meling T.R. (2013). Intraventricular meningiomas: A consecutive series of 22 patients and literature review. Neurosurg. Rev..

[10] Konglund A., Rogne S.G., Helseth E., Meling T.R. (2013). Meningioma surgery in the very old-validating prognostic scoring systems. Acta Neurochir..

[11] Meling T.R., Da Broi M., Scheie D., Helseth E. (2019). Skull base versus non-skull base meningioma surgery in the elderly. Neurosurg. Rev..

[12] Combs S.E., Ganswindt U., Foote R.L., Kondziolka D., Tonn J.C. (2013). State-of-the-art treatment alternatives for base of skull meningiomas: Complementing and controversial indications for neurosurgery, stereotactic and robotic based radiosurgery or modern fractionated radiation techniques. Radiat. Oncol..

[13] Faramand A., Kano H., Niranjan A., Johnson S.A., Hassib M., Park K.J., Arai Y., Flickinger J.C., Lunsford L.D. (2018). Cranial nerve outcomes after primary stereotactic radiosurgery for symptomatic skull base meningiomas. J. Neurooncol..

[14] Komotar R.J., Starke R.M., Raper D.M., Anand V.K., Schwartz T.H. (2012). Endoscopic endonasal versus open transcranial resection of anterior midline skull base meningiomas. World Neurosurg..

[15] Schneider M., Schuss P., Guresir A., Borger V., Vatter H., Guresir E. (2020). Surgery for posterior fossa meningioma: Elevated postoperative cranial nerve morbidity discards aggressive tumor resection policy. Neurosurg. Rev..

[16] Schneider M., Schuss P., Guresir A., Wach J., Hamed M., Vatter H., Guresir E. (2019). Cranial Nerve Outcomes After Surgery for Frontal Skull Base Meningiomas: The Eternal Quest of the Maximum-Safe Resection with the Lowest Morbidity. World Neurosurg..

[17] Goldsmith B.J., Wara W.M., Wilson C.B., Larson D.A. (1994). Postoperative irradiation for subtotally resected meningiomas. A retrospective analysis of 140 patients treated from 1967 to 1990. J. Neurosurg..

[18] Mathiesen T., Lindquist C., Kihlstrom L., Karlsson B. (1996). Recurrence of cranial base meningiomas. Neurosurgery.

[19] Materi J., Mampre D., Ehresman J., Rincon-Torroella J., Chaichana K.L. (2020). Predictors of recurrence and high growth rate of residual meningiomas after subtotal resection. J. Neurosurg..

[20] Worldometer.info Worldometer. www.worldometer.info.

[21] Brokinkel B., Holling M., Spille D.C., Hess K., Sauerland C., Bleimuller C., Paulus W., Wolfer J., Stummer W. (2016). Surgery for meningioma in the elderly and long-term survival: Comparison with an age- and sex-matched general population and with younger patients. J. Neurosurg..

[22] Cahill K.S., Claus E.B. (2011). Treatment and survival of patients with nonmalignant intracranial meningioma: Results from the Surveillance, Epidemiology, and End Results Program of the National Cancer Institute. Clinical article. J. Neurosurg..

[23] Park J.S., Sade B., Oya S., Kim C.G., Lee J.H. (2014). The influence of age on the histological grading of meningiomas. Neurosurg. Rev..

[24] Kallio M., Sankila R., Hakulinen T., Jaaskelainen J. (1992). Factors affecting operative and excess long-term mortality in 935 patients with intracranial meningioma. Neurosurgery.

[25] Gerlach R., Raabe A., Scharrer I., Meixensberger J., Seifert V. (2014). Post-operative hematoma after surgery for intracranial meningiomas: Causes, avoidable risk factors and clinical outcome. Neurol. Res..

[26] Lassen B., Helseth E., Ronning P., Scheie D., Johannesen T.B., Maehlen J., Langmoen I.A., Meling T.R. (2011). Surgical mortality at 30 days and complications leading to recraniotomy in 2630 consecutive craniotomies for intracranial tumors. Neurosurgery.

[27] Roser F., Ebner F.H., Ritz R., Samii M., Tatagiba M.S., Nakamura M. (2007). Management of skull based meningiomas in the elderly patient. J. Clin. Neurosci..

[28] Kressner M., Arlt F., Riepl W., Meixensberger J. (2018). Prognostic factors of microsurgical treatment of intracranial meningiomas-A multivariate analysis. PLoS ONE.

[29] Van Alkemade H., de Leau M., Dieleman E.M., Kardaun J.W., van Os R., Vandertop W.P., van Furth W.R., Stalpers L.J. (2012). Impaired survival and long-term neurological problems in benign meningioma. Neuro Oncol..

[30] Caroli M., Locatelli M., Prada F., Beretta F., Martinelli-Boneschi F., Campanella R., Arienta C. (2005). Surgery for intracranial meningiomas in the elderly: A clinical-radiological grading system as a predictor of outcome. J. Neurosurg..

[31] Cohen-Inbar O., Soustiel J.F., Zaaroor M. (2009). Meningiomas in the elderly, the surgical benefit and a new scoring system. Acta Neurochir..

[32] Cohen-Inbar O., Sviri G.E., Soustiel J.F., Zaaroor M. (2011). The Geriatric Scoring System (GSS) in meningioma patients--validation. Acta Neurochir..

[33] Samii M., Klekamp J., Carvalho G. (1996). Surgical results for meningiomas of the craniocervical junction. Neurosurgery.

[34] Levine Z.T., Buchanan R.I., Sekhar L.N., Rosen C.L., Wright D.C. (1999). Proposed grading system to predict the extent of resection and outcomes for cranial base meningiomas. Neurosurgery.

[35] Mansouri A., Klironomos G., Taslimi S., Kilian A., Gentili F., Khan O.H., Aldape K., Zadeh G. (2016). Surgically resected skull base meningiomas demonstrate a divergent postoperative recurrence pattern compared with non-skull base meningiomas. J. Neurosurg..

[36] (2011). Roberti F, Sekhar LN, Kalavakonda C, Wright DC Posterior fossa meningiomas-surgical experience in 161 cases. Surg. Neurol..

[37] Gallagher M.J., Jenkinson M.D., Brodbelt A.R., Mills S.J., Chavredakis E. (2016). WHO grade 1 meningioma recurrence: Are location and Simpson grade still relevant?. Clin. Neurol. Neurosurg..

[38] Ildan F., Erman T., Gocer A.I., Tuna M., Bagdatoglu H., Cetinalp E., Burgut R. (2007). Predicting the probability of meningioma recurrence in the preoperative and early postoperative period: A multivariate analysis in the midterm follow-up. Skull Base.

[39] Kollová A., Liscák R., Novotný J., Vladyka V., Simonová G., Janousková L. (2007). Gamma Knife surgery for benign meningioma. J. Neurosurg..

[40] Kondziolka D., Mathieu D., Lunsford L.D., Martin J.J., Madhok R., Niranjan A., Flickinger J.C. (2008). Radiosurgery as definitive management of intracranial meningiomas. Neurosurgery.

[41] Stafford S.L., Pollock B.E., Foote R.L., Link M.J., Gorman D.A., Schomberg P.J., Leavitt J.A. (2001). Meningioma radiosurgery: Tumor control, outcomes, and complications among 190 consecutive patients. Neurosurgery.

[42] Ostrom Q.T., Gittleman H., Xu J., Kromer C., Wolinsky Y., Kruchko C., Barnholtz-Sloan J.S. (2016). CBTRUS Statistical Report: Primary Brain and Other Central Nervous System Tumors Diagnosed in the United States in 2009-2013. Neuro Oncol..

[43] Louis D.N., Perry A., Reifenberger G., von Deimling A., Figarella-Branger D., Cavenee W.K., Ohgaki H., Wiestler O.D., Kleihues P., Ellison D.W. (2016). The 2016 World Health Organization Classification of Tumors of the Central Nervous System: A summary. Acta Neuropathol..

[44] Landriel Ibanez F.A., Hem S., Ajler P., Vecchi E., Ciraolo C., Baccanelli M., Tramontano R., Knezevich F., Carrizo A. (2011). A new classification of complications in neurosurgery. World Neurosurg..

